# Beyond performance metrics: modeling outcomes and cost for clinical machine learning

**DOI:** 10.1038/s41746-021-00495-4

**Published:** 2021-08-10

**Authors:** James A. Diao, Leia Wedlund, Joseph Kvedar

**Affiliations:** 1 Harvard Medical School, Boston, MA USA; 2 Mass General Brigham, Boston, MA USA

**Keywords:** Health care economics, Computational science, Outcomes research

## Abstract

Advances in medical machine learning are expected to help personalize care, improve outcomes, and reduce wasteful spending. In quantifying potential benefits, it is important to account for constraints arising from clinical workflows. Practice variation is known to influence the accuracy and generalizability of predictive models, but its effects on cost-effectiveness and utilization are less well-described. A simulation-based approach by Mišić and colleagues goes beyond simple performance metrics to evaluate how process variables may influence the impact and financial feasibility of clinical prediction algorithms.

Advances in medical machine learning are expected to help personalize care, improve outcomes, and reduce wasteful spending. In quantifying potential benefits, it is important to account for constraints arising from clinical workflows. Practice variation is known to influence the accuracy and generalizability of predictive models^1,2^, but its effects on cost-effectiveness and utilization are less well-described. A simulation-based approach by Mišić and colleagues^3^ goes beyond simple performance metrics to evaluate how process variables may influence the impact and financial feasibility of clinical prediction algorithms.

Mišić et al.’s study builds on previous work that developed equations for predicting unplanned readmissions^4^. Readmission rate is a publicly reported metric that commands significant attention in quality improvement and cost management initiatives. As part of these efforts, prediction equations are used to stratify readmission risk and allocate limited interventions to the patients who need it most. Still, many important questions—how many interventions are applied, how many readmissions are prevented, and how much spending is averted—are often unclear.

Mišić and colleagues provide answers by simulating patient flow for a hypothetical clinical workflow. Under their model, each patient is assigned a risk score based on four separate algorithms (LACE, HOSPITAL, and two locally-designed equations) for each day a prediction is available. On each day that a provider is available, the eight highest-risk patients are “treated” with a hypothetical intervention that has a 10% chance of preventing readmission. The authors applied this model to a dataset of 19,331 post-operative surgical patients from the UCLA Ronald Reagan Medical Center, including 969 (5.0%) who were later readmitted. Because the described workflow is speculative, it cannot be validated with data. Instead, the design and parameters were chosen to reflect typical staff and time constraints.

Using these simulation conditions, Mišić and colleagues compute utilization and volume metrics for the Ronald Reagan Medical Center, including interventions conducted, readmissions anticipated, and expected readmissions prevented. To compute net savings, the authors added the highest-cost ICD-10 codes for each “prevented” readmission and subtracted expected labor costs. By toggling simulation parameters, the authors also show how differences in accuracy, prediction timing, and provider availability translate into differences in outcomes. For example, algorithms that rely on length of stay are unable to assign risk scores before the day of discharge, potentially constraining opportunities to intervene. The authors also find several parameter settings where costs outweigh savings, consistent with earlier studies showing that interventions for preventing readmission are not ﻿always cost-effective^5^.

The simulation approach relies on a broad set of simplifying assumptions and therefore has several limitations. In particular, the assumption of fixed, limited availability (e.g., one nurse practitioner providing readmission interventions for a 520-bed hospital) may be overly stringent, or may overlook the need for additional funding to support effective programs. Assumptions for intervention timing may also be inexact, as strategies for preventing hospital readmission increasingly comprise multiple components administered before, during, and after discharge^6^. Last, the evaluated algorithms do not account for many important drivers of readmissions, such as language and cultural barriers, mental illness, and poverty. Allocating interventions based on clinical risk alone may not represent the most common or effective strategy for reducing rehospitalization. Together, these considerations indicate the need to validate simulation results against real-world data and recalibrate assumptions where necessary. Beyond validation, future work should extend the model to provide estimates of uncertainty and evaluate health and equity-based outcomes.

Ultimately, the proposed simulation model provides estimates for utilization and financial feasibility in the setting of a specific clinical workflow. Preventing rehospitalization is only one application; others include prevention of sepsis^7^ or acute kidney injury^8^. While not a substitute for randomized trials^9^, simulation modeling can provide initial answers and insights for all stakeholders involved, including researchers developing prediction algorithms, administrators optimizing clinical workflows, executives evaluating business models, and regulators seeking to understand performance in context.

For decades, medical practice has proved impervious to algorithmic reinvention^10^. One contributor is imperfect communication of a clear value proposition centered on outcomes, costs, and metrics that matter. Performance metrics like sensitivity and specificity are only part of the puzzle. A simulated modeling approach may help contextualize and complement traditional accuracy metrics to strengthen the case for new prediction models.
